# Microbiome and Metagenome Analyses of a Closed Habitat during Human Occupation

**DOI:** 10.1128/mSystems.00367-20

**Published:** 2020-07-28

**Authors:** Ganesh Babu Malli Mohan, Ceth W. Parker, Camilla Urbaniak, Nitin K. Singh, Anthony Hood, Jeremiah J. Minich, Rob Knight, Michelle Rucker, Kasthuri Venkateswaran

**Affiliations:** a Biotechnology and Planetary Protection Group, Jet Propulsion Laboratory, California Institute of Technology, Pasadena, California, USA; b Exploration Integration and Science Directorate, NASA Johnson Space Center, Houston, Texas, USA; c Marine Biology Research Division, Scripps Institute of Oceanography, University of California San Diego, La Jolla, California, USA; d Departments of Pediatrics, Bioengineering, and Computer Science & Engineering, and Center for Microbiome Innovation, University of California San Diego, La Jolla, California, USA; Cleveland Clinic

**Keywords:** extravehicular activity, Analog habitat, microbiome, microbial diversity, functional metagenomics, spacecraft microbiome, closed habitat, metagenomics

## Abstract

Microbial contamination during long-term confinements of space exploration presents potential risks for both crew members and spacecraft life support systems. A novel swab kit was used to sample various surfaces from a submerged, closed, analog habitat to characterize the microbial populations. Samples were collected from various locations across the habitat which were constructed from various surface materials (linoleum, dry wall, particle board, glass, and metal), and microbial populations were examined by culture, quantitative PCR (qPCR), microbiome 16S rRNA gene sequencing, and shotgun metagenomics. Propidium monoazide (PMA)-treated samples identified the viable/intact microbial population of the habitat. The cultivable microbial population ranged from below the detection limit to 10^6^ CFU/sample, and their identity was characterized using Sanger sequencing. Both 16S rRNA amplicon and shotgun sequencing were used to characterize the microbial dynamics, community profiles, and functional attributes (metabolism, virulence, and antimicrobial resistance). The 16S rRNA amplicon sequencing revealed abundance of viable (after PMA treatment) *Actinobacteria* (*Brevibacterium*, *Nesternkonia*, *Mycobacterium*, *Pseudonocardia*, and *Corynebacterium*), *Firmicutes* (*Virgibacillus*, *Staphylococcus*, and *Oceanobacillus*), and *Proteobacteria* (especially Acinetobacter) on linoleum, dry wall, and particle board (LDP) surfaces, while members of *Firmicutes* (*Leuconostocaceae*) and *Proteobacteria* (*Enterobacteriaceae*) were high on the glass/metal surfaces. Nonmetric multidimensional scaling determined from both 16S rRNA and metagenomic analyses revealed differential microbial species on LDP surfaces and glass/metal surfaces. The shotgun metagenomic sequencing of samples after PMA treatment showed bacterial predominance of viable *Brevibacterium* (53.6%), *Brachybacterium* (7.8%), *Pseudonocardia* (9.9%), *Mycobacterium* (3.7%), and *Staphylococcus* (2.1%), while fungal analyses revealed *Aspergillus* and *Penicillium* dominance.

**IMPORTANCE** This study provides the first assessment of monitoring cultivable and viable microorganisms on surfaces within a submerged, closed, analog habitat. The results of the analyses presented herein suggest that the surface material plays a role in microbial community structure, as the microbial populations differed between LDP and metal/glass surfaces. The metal/glass surfaces had less-complex community, lower bioburden, and more closely resembled the controls. These results indicated that material choice is crucial when building closed habitats, even if they are simply analogs. Finally, while a few species were associated with previously cultivated isolates from the International Space Station and MIR spacecraft, the majority of the microbial ecology of the submerged analog habitat differs greatly from that of previously studied analog habitats.

## Abstract (precis)

This study provides the first assessment of monitoring cultivable and viable microorganisms on surfaces within a submerged, closed, analog habitat. The results of the analyses presented herein suggest that the surface material plays a role in microbial community structure, as the microbial populations differed between LDP and metal/glass surfaces. The metal/glass surfaces had less-complex community, lower bioburden, and more closely resembled the controls. These results indicated that material choice is crucial when building closed habitats, even if they are simply analogs. Finally, while a few species were associated with previously cultivated isolates from the International Space Station and MIR spacecraft, the majority of the microbial ecology of the submerged analog habitat differs greatly from that of previously studied analog habitats.

## INTRODUCTION

Over the next few decades, the National Aeronautics and Space Administration (NASA), along with international partners, has planned to begin the expansion of human space exploration beyond low Earth orbit, to the moon and on to Mars. This endeavor will entail a variety of new space habitats designed for both interplanetary travel and surface habitation (1, 2). Minimizing and monitoring the number of detrimental microorganisms associated with these habitats will be critical to the safety and success of these missions. Inevitably, the components that will lead to the majority of the contamination of these facilities are the human occupants (3, 4). Indeed, humans host an intricate microbiome consisting of numerous microorganisms that live on and within us (5). Most of these microorganisms are either benign and pose no danger to the inhabitant or are beneficial and perform necessary roles like protection from pathogens or conversion of nutrients into more readily absorbed compounds in our gut. However, humans can also unknowingly harbor, transport, and disperse pathogens and other microorganisms that can lead to microbially induced corrosion (MIC; also known as biocorrosion) (6). While the presence of microbial contaminants with either pathogenic traits or MIC capabilities will pose a direct threat to the closed habitat and the crew, it is impractical and unrealistic to completely remove all microorganisms. Thus, similar to recent trends in hospital and medical instrument design, engineering mechanisms into habitat construction to manage microbial ecology while continuously monitoring the remaining microbial populations will be critical to future space mission success (7, 8).

To aid NASA in developing appropriate closed habitats (a built environment that has minimal atmospheric exchange with the surrounding environment), it will be necessary to characterize and compare the microbial ecology across a variety of closed environments. In this regard, microbial analysis of several closed systems has been performed, including the spacecraft assembly facilities (SAF) (9), Inflated Lunar/Mars Analogous Habitat (ILMAH) (10, 11), long haul commercial aircraft cabin air (12), and the International Space Station (ISS) (13–15). However, this is the first report to characterize the molecular and microbial communities of a submerged analog habitat. The analog habitat tested during this study mimics the ISS in that it is a pressure vessel closed off from the surrounding environment where a crew lives in isolation, similar to what would be experienced during a 3-week-long space mission.

In the past, samples from built environments and spacecraft were collected using swabs made up of cotton, rayon, and polyester swabs (16, 17). Recently, new sampling devices were tested for microbial sampling; this includes upgraded swabs (different materials), polyester wipes, macrofoam sponges, adhesive tapes, biological sampling kit (BiSKit; macrofoam), witness coupons, dust, and bulk sampling (16, 18–24). These novel microbial sampling tools have allowed for much more comprehensive collection of both cultivable and yet to be cultivated microbial populations (17, 23, 25). Furthermore, these sampling devices were also employed to collect samples from Earth-based analogs (Mars 500 facility/ILMAH/Antarctic Concordia Station), and the ISS (11, 13, 14, 26–28).

Engineers and scientists from the Human Forward Contamination Assessment team at NASA’s Johnson Space Center (JSC) have recently developed a novel sampling device, extravehicular activity (EVA) microbial swab kit (29). This swab kit consists of a macrofoam paddle head held in a large tool handle and stored in an eight-canister sample caddy, which is designed for astronauts to use while wearing cumbersome EVA space suit gloves. In addition to this kit being designed for sampling outside surfaces of the ISS and spacesuits, initial evaluations demonstrated that these EVA swabs outperformed standard swabs in their ability to pick up microbial cells (29). These evaluations demonstrated the ability of the EVA swabs to successfully collect spores and cells from sample surfaces.

The objective of this study was to characterize the microbial community of a submerged analog habitat. Using the previously validated EVA swab kit, 12 samples were collected from various surface materials (linoleum, particle board, dry wall, glass, and metal surfaces). Traditional microbiological methods and molecular techniques were employed to uncover the microbial diversity of this analog habitat. In order to understand the viable microbial population, samples were pretreated with propidium monoazide (PMA), a chemical that chelates free DNA within dead cells and prevents its amplification during PCR (30). This approach allows the viable/intact microbial community to be differentiated from dead cells. The 16S rRNA gene amplicon and shotgun metagenome sequencing were performed to characterize the microbial diversity as well as functional pathways of the viable microbiome.

## RESULTS

Among the locations sampled (*n* = 12) and controls (*n* = 4), there was distinctive grouping noticed based on material type. The results of traditional microbiological and molecular methods were computed, and both microbial burden and microbial diversity were differentially distributed between the material types. The statistically significant *P* values are given in the respective sections. A summary of the locations sampled and their respective material surface type is presented in Table 1.

**TABLE 1:** Description of the sampling locations of the Analog habitat and its description

EVA swab	Sampling location used in this study	Sampling location description	Surface material	Site category
EE101	E1	Swab control	Swab	Control
EE102	E2	Air control	Swab	Control
EE103	E3	Center of view port window in the bunk area	Glass	Metal/glass
EE104	E4	Floor between bunks	Particle board	LDP
EE105	E5	Counter top by the phone	Metal	Metal/glass
EE106	E6	Galley table top	Metal	Metal/glass
EE107	E7	Floor in front of the galley sink	Particle board	LDP
EE108	E8	Science table top in the entry lock	Metal	Metal/glass
EE109	E9	Swab control	Swab	Control
EE110	E10	Air control	Swab	Control
EE111	E11	Behind the entry lock sink	Metal	Metal/glass
EE112	E12	Entry lock floor	Particle board	LDP
EE113	E13	Wall above the waste/hygiene compartment (WHC)	Dry wall	LDP
EE114	E14	Wet porch table top	Metal	Metal/glass
EE115	E15	Bottom of the top bunk above the pillow	Metal	Metal/glass
EE116	E16	Trash can storage place	Linoleum	LDP

### Estimation of cultivable microbial burden.

**FIG 1:**
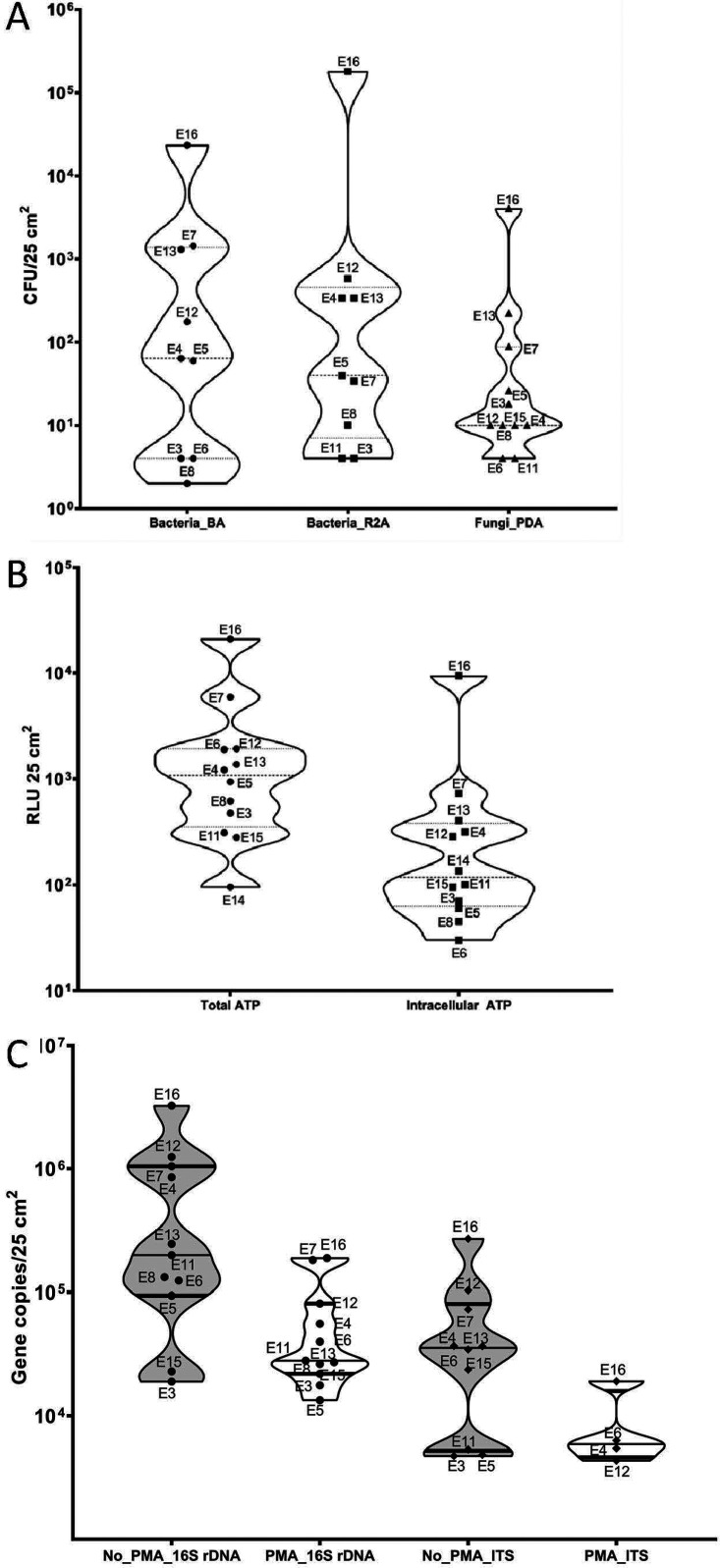
Culture-dependent and -independent analysis of Analog habitat surface samples. (A) Abundance of cultivable bacteria and fungi. Each dot in a column represents Analog habitat location sampled. No statistically significant differences in abundances were observed among flight missions and between bacteria and fungi (one-way ANOVA, *P* > 0.01). (B) The relative light unit (RLU) values for ATP counts for total ATP (small circles) and intracellular ATP (small squares). No statistically significant differences in abundances were observed among flight missions and between bacteria and fungi (one-way ANOVA, *P* > 0.01). (C) The qPCR-based microbial burden (total; non-PMA and viable; PMA) of various Analog habitat surface sample. The gene copies were measured by targeting 16S rRNA gene (bacteria) and ITS gene (fungi).

The microbial populations of various surfaces from within the Analog habitat estimated by the culture-dependent CFU counts and the culture-independent methods of quantitative PCR (qPCR) and ATP assays are given in Table 2. Cultivable population (mesophiles on Reasoner’s 2A agar [R2A] medium) from the Analog habitat surfaces ranged from 4.0 × 10^0^ to 1.8 × 10^5^ CFU/25 cm^2^ except in samples collected from location E14 (wet porch table top) or location E15 (bottom of top bunk above pillow). Similarly, when Analog habitat surface samples were grown on blood agar (BA) plates, the microbial population ranged from 4.0 × 10^0^ to 2.3 × 10^4^ CFU/25 cm^2^ except in samples collected from locations E14, E15, and E6 (galley table top). CFU were statistically significantly higher in the Analog habitat linoleum, dry wall, and particle board (LDP) samples (E4, E7, E12, E13, and E16) than in the metal/glass surface samples (E3, E6, E8, and E11) (*P* value of <0.01). The cultivable fungal population measured on potato dextrose agar (PDA) plates ranged from 4.0 × 10^0^ to 4.0 × 10^4^ CFU/25 cm^2^, with no growth observed from E14 (Fig. 1A). Notably, locations E7, E13, and E16 (LDP material types) harbored higher fungal populations compared to the other locations (metal/glass); however, statistical analysis did not show any significance. In general, average cultivable fungi were ∼2 log units less than the average bacterial population tested in all samples. All four controls tested (samples E1, E2, E9, and E10) yielded no bacterial (R2A or BA media) or fungal isolates.

**TABLE 2:** Total, viable, and cultivable microbiological characteristics of the samples collected from the Analog habitat surfaces

Sample	Cultivable bacterial population (CFU/25 cm^2^)^*a*^		qPCR-based bacterial population (no. of 16S rRNA copies 25 cm^2^)		Viable bacterial population (*B*/*A* × 100)	ATP-based microbial population (RLU/25 cm^2^)^*b*^		Viable microbial population (*D*/*C* × 100)
	Bacteria	Fungi	Untreated (*A*)	PMA-treated (*B*)		Total ATP (*C*)	Intracellular ATP (*D*)	
E1	NG	NG	BDL	BDL		BDL	BDL	
E2	NG	NG	BDL	BDL		BDL	BDL	
E3	4.0	1.8 × 10^1^	1.90 × 10^4^	1.77 × 10^4^	93.32	4.76 × 10^2^	7.00 × 10^1^	14.71
E4	4.0 × 10^2^	1.0 × 10^1^	8.52 × 10^5^	5.57 × 10^4^	6.54	1.22 × 10^3^	3.15 × 10^2^	25.92
E5	1.0 × 10^2^	2.6 × 10^1^	9.39 × 10^4^	1.34 × 10^4^	14.23	9.40 × 10^2^	6.00 × 10^1^	6.38
E6	4.0	4.0	1.24 × 10^5^	3.99 × 10^4^	32.23	1.93 × 10^3^	3.00 × 10^1^	1.55
E7	1. 5 × 10^3^	8.8 × 10^1^	1.05 × 10^6^	1.81 × 10^5^	17.34	5.92 × 10^3^	7.32 × 10^2^	12.36
E8	2.0	1.0 × 10^1^	1.32 × 10^5^	2.63 × 10^4^	19.92	6.20 × 10^2^	4.50 × 10^1^	7.29
E9	NG	NG	BDL	BDL		BDL	BDL	
E10	NG	NG	BDL	BDL		BDL	BDL	
E11	4.0	4.0	1.99 × 10^5^	2.80 × 10^4^	14.07	2.80 × 10^2^	9.52 × 10^1^	34.00
E12	7.6 × 10^2^	1.0 × 10^1^	1.25 × 10^6^	8.11 × 10^4^	6.51	1.9 × 10^3^	2.85 × 10^2^	15.04
E13	1.6 × 10^3^	2.2 × 10^2^	2.46 × 10^5^	2.72 × 10^4^	11.09	1.37 × 10^3^	4.04 × 10^2^	29.45
E14	NG	NG	2.69 × 10^4^	2.38 × 10^4^	88.54	9.52 × 10^1^	1.35 × 10^2^	>100
E15	NG	1.0 × 10^1^	2.29 × 10^4^	2.19 × 10^4^	95.47	3.10 × 10^2^	1.00 × 10^2^	32.26
E16	2.0 × 10^5^	4.0 × 10^3^	3.25 × 10^6^	1.88 × 10^5^	5.79	2.09 × 10^4^	9.40 × 10^3^	44.93

a NG, no growth.

b The ATP standard curve was generated by the method of Benardini and Venkateswaran (89) with 50 RLU = 1.169 × 10^−11^ mmol of ATP.

### Estimation of molecular microbial burden.

The total ATP (tATP) value measured both live and dead microbial cells and ranged from below the detection limit (BDL) to 2.09 × 10^4^ RLU (relative luminescence unit) per 25 cm^2^, whereas intracellular ATP (iATP) values (which measures only viable cells) ranged from BDL to 9.40 × 10^3^ RLU per 25 cm^2^. The iATP values were highest from E7 (floor in front of galley sink), E13 (wall above the waste/hygiene compartment [WHC]), and E16 (trash can storage place) (Fig. 1B). In general, the viable microbial burden as measured by the iATP content was highest in LDP surface materials and lowest in metal/glass surface materials (Table 2), but overall, there was no statistical significance between these two groups.

The qPCR assay revealed that the non-PMA-treated samples (which contains live and dead cells) had 16S rRNA gene copy numbers that ranged from BDL to 3.25 × 10^6^ copies per 25 cm^2^, whereas the PMA-treated samples (viable/intact cells) had 16S gene copy numbers that range from BDL to 1.88 × 10^5^ copies per 25 cm^2^. The average 16S rRNA gene copy number from the PMA-treated group was 5.87 × 10^4^ copies 25 cm^2^. Notably, more viable bacteria (>5 × 10^4^ copies per 25 cm^2^) were detected from LDP surfaces (E4, E7, E12, and E16) than from the metal/glass surfaces (Fig. 1C, PMA_16S rRNA). This is congruent with the culture data where LDP samples had higher CFU counts than the metal/glass samples (*P* < 0.01). The total fungal internal transcribed spacer (ITS) gene copy number of non-PMA-treated samples ranges from BDL to 6.75 × 10^5^ copies 25 cm^2^, whereas PMA-treated ITS gene copy number ranges from BDL to 4.77 × 10^4^ copies 25 cm^2^. The average fungal ITS gene copy number was 2.2 × 10^4^ copies per sample. The highest viable fungal burden was found at locations E4, E6, E12, and E16, while all other locations were BDL (Fig. 1C, PMA_ITS). Overall, both culture-dependent and -independent methods demonstrated highest microbial bioburden at locations E7 and E16, both belonging to the LDP category.

### Cultivable microbial diversity.

The 16S rRNA and ITS amplicons identified via Sanger sequencing and phylogenetic affiliation of the bacterial and fungal strains isolated in this study are shown in Fig. S1 and S2 in the supplemental material. A total of 33 bacterial and 47 fungal strains were isolated from the analog habitat surfaces and identified. The bacterial isolates belonged to the phyla *Actinobacteria*, *Firmicutes*, and *Proteobacteria*. At the genus level, the most predominant genera were *Bacillus* and *Staphylococcus*, comprising 72% and 15% of the isolates identified, respectively. At the species level, the most abundant was Bacillus zhangzhouensis (comprising 42.4% of total bacterial isolates; Fig. S1). The fungal isolates were mostly represented by Aspergillus sydowii and *Ascomycota* species, comprising 36% and 17% of total fungal isolates, respectively (Fig. S2).

10.1128/mSystems.00367-20.1 FIG S1 Checkerboard plot and phylogenetic tree based on 16S rRNA sequences of various bacterial strains isolated from the Analog habitat. Download FIG S1, PDF file, 0.1 MB. Copyright © 2020 Malli Mohan et al. 2020 Malli Mohan et al. This content is distributed under the terms of the Creative Commons Attribution 4.0 International license.

10.1128/mSystems.00367-20.2 FIG S2 Checkerboard plot and phylogenetic tree based on 16S rRNA sequences of various fungal strains isolated from the Analog habitat. Download FIG S2, PDF file, 0.1 MB. Copyright © 2020 Malli Mohan et al. 2020 Malli Mohan et al. This content is distributed under the terms of the Creative Commons Attribution 4.0 International license.

### Analysis of bacteria.

**FIG 2:**
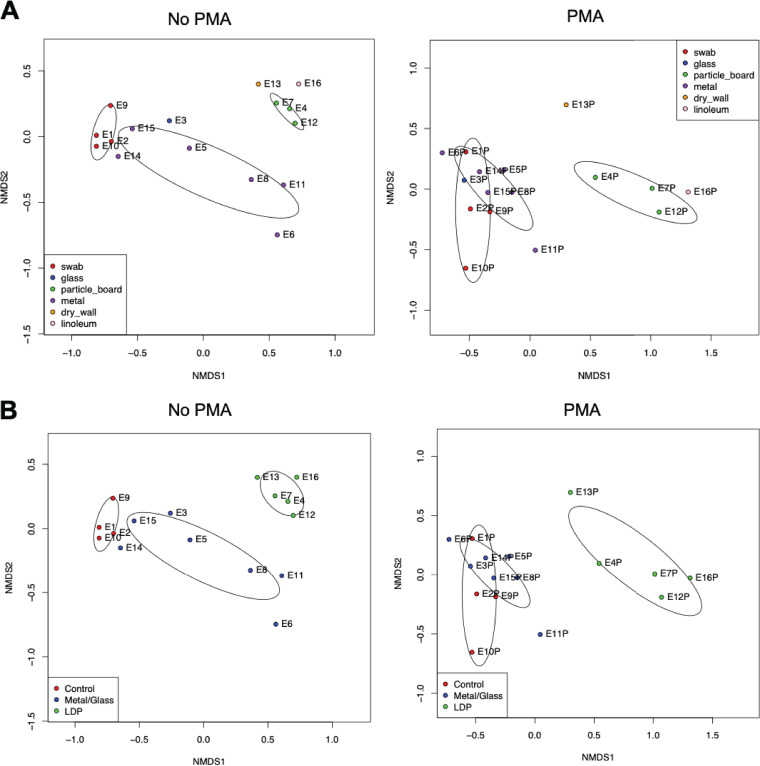
Multidimensional scaling plots of 16S rRNA gene amplicon sequencing data. NMDS ordination showing 99% confidence interval ellipses of non-PMA-treated (left panels) and PMA-treated (right panels) grouped based on surface material (A) and site categories (B). The various samples collected from across the Analog habitat are indicated by dots, and the closer the dots are to each other, the more similar their bacterial composition is in terms of types and number of bacteria.

Nonmetric multidimensional scaling (NMDS) plots were computed using Bray-Curtis dissimilarity calculations to compare beta diversity (microbial composition and abundance) among the different samples. Similar trends in sample groupings were observed between non-PMA- and PMA-treated samples, except for the wall sample which had a distinct microbiome among the PMA-treated samples (Fig. 2A, right panel) but which was closely related to the particle board samples in the non-PMA-treated group (Fig. 2A, left panel). The linoleum sample was also similar to the particle board samples in both PMA- and non-PMA-treated groups (Fig. 2A). Figure 2B is the same NMDS plot as Fig. 2A, but statistically analyzing the difference between surface material, which clearly show that the microbiome of the metal/glass samples is different from the microbiome of the water/floor samples. Permutational multivariate analysis of variance (PERMANOVA) confirms the separation of the groups seen in the NMDS plots with *P* values of <0.01.

A variety of bacteria were detected by 16S rRNA gene amplicon sequencing, belonging to seven phyla, with 5% of the overall reads not able to be assigned to a phylum (Table 3). A total of 1,354,846 reads were generated from the non-PMA-treated samples, while 133,843 reads were generated from the PMA-treated samples, suggesting that majority of the reads were from dead cells (Table 3) with viable bacteria making up 10% of the total bacterial community. This was true regardless of surface material. LDP samples (*n* = 5) had ∼7× more reads compared to the metal/glass surfaces, which was true for both PMA- and non-PMA-treated samples (Table 3). Alpha diversity using Shannon’s index was performed, and the average index for PMA-treated LDP samples was 2.86, and for metal/glass, it was 2.13. A Student’s *t* test showed that these groups were not statistically significant. For the non-PMA-treated samples, the average indexes were 2.52 for LDP and 3.1 for metal/glass samples, and these were also not statistically different.

**TABLE 3:** Bacterial phyla retrieved from various surfaces of the Analog habitat

Taxon	No. of 16S rRNA amplicon reads from samples:			
	No PMA, LDP	PMA, LDP	No PMA, metal/glass	PMA, metal/glass
*Actinobacteria*	852,891	71,011	16,611	757
*Bacteroidetes*	2,444	190	58	
*Firmicutes*	115,140	21,236	36,816	10,446
*Fusobacteria*			3,119	
*Alphaproteobacteria*	58,475	4,003	9,467	3,597
*Betaproteobacteria*	1,240	2,288	3,694	4,753
*Gammaproteobacteria*	105,666	8,176	76,384	3,259
Unassigned	41,008	4,127	31,833	

Total no. of reads	1,176,864	111,031	177,982	22,812

**FIG 3:**
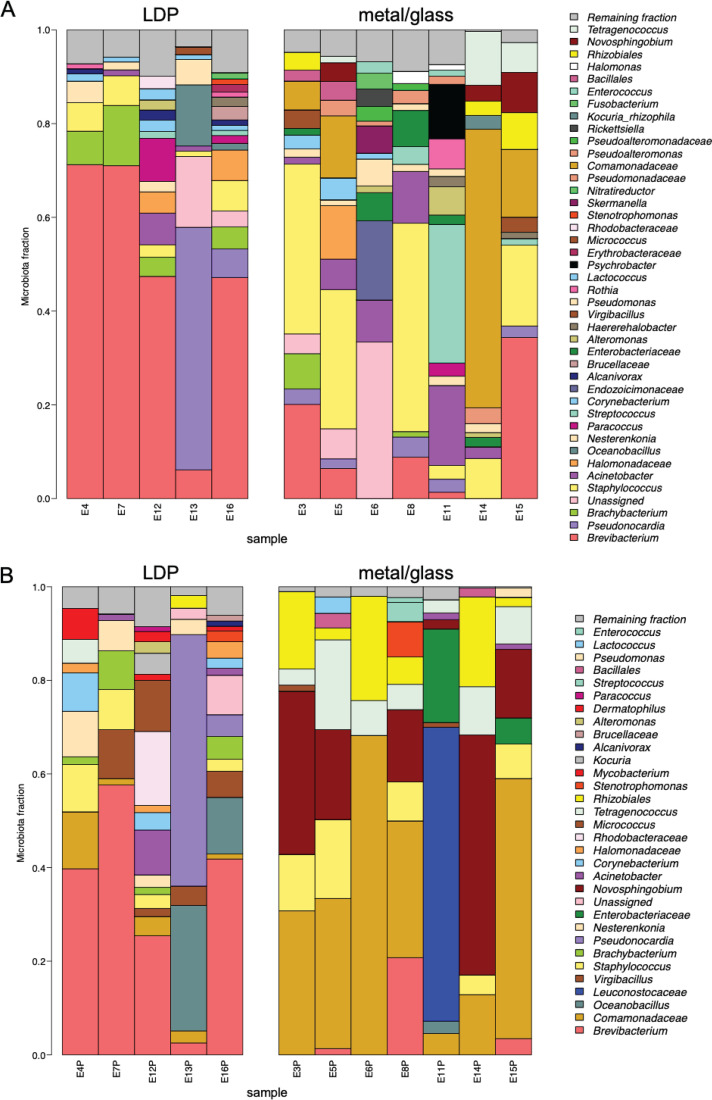
Relative abundances of bacteria detected by 16S rRNA gene amplicon sequencing. The relative abundances of bacterial taxa identified in various samples across the Analog habitat were visualized by bar plots. Each bar represents a specific sample, and each colored box represents a particular taxon. The height of the colored box represents the relative abundance of that particular taxon within the sample. Taxa present in less than 1% abundance in a given sample are displayed in the “remaining fraction” at the top of the graph (gray box). The legend is read from bottom to top, with the bottom taxon in the legend corresponding to the bottom taxon on the graph. Non-PMA treated samples are displayed in panel A, and the PMA-treated samples are displayed in panel B.

In total, when classified reads were summarized to the genus level, 52 bacterial genera were identified (see Data Set S1 in the supplemental material) with the proportions found in each sample summarized in Fig. 3. The five most abundant genera detected in the non-PMA-treated group were *Brevibacterium*, *Pseudonocardia*, *Brachybacterium*, *Staphylococcus*, and Acinetobacter (Fig. 3A) while the five most abundant genera detected in the PMA-treated group belong to the members of *Brevibacterium*, *Comamonadaceae*, *Oceanobacillus*, *Leuconostocaceae*, and *Virgibacillus* (Fig. 3B).

10.1128/mSystems.00367-20.5 DATA SET S1 Table of reads of amplicon sequences summarized to the genus level. Download Data Set S1, XLSX file, 0.02 MB. Copyright © 2020 Malli Mohan et al. 2020 Malli Mohan et al. This content is distributed under the terms of the Creative Commons Attribution 4.0 International license.

**FIG 4:**
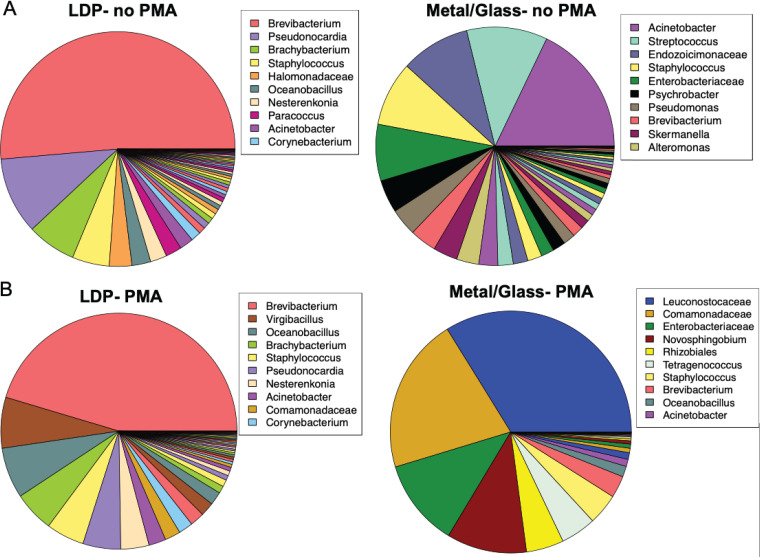
Differential bacterial composition among various types of surfaces. Pie chart of the relative abundances of bacteria detected in the Analog habitat. The sequences obtained were summarized to the genus level. In total, 52 taxa were detected, but only the 10 most abundant taxa are displayed in the legends. The pie graphs are separated based on the site categories: LDP samples (left panels) and metal/glass (right panels) and treatment groups, no PMA (A) and PMA (B).

There were also differences in the bacterial composition among the LDP and metal/glass samples with LDP having a high proportion of *Brevibacterium*, *Pseudonocardia*, *Brachybacterium*, and *Halomonadaceae*. In contrast, metal/glass samples had a high proportion of Acinetobacter, *Streptococcus*, *Endozoicimonaceae*, *Enterobacteriaceae*, and *Psychrobacter* for the non-PMA-treated group (Fig. 4A). For the PMA-treated group, the LDP samples had a higher proportion of *Brevibacterium*, *Virgibacillus*, *Oceanbacillus*, *Brachybacterium*, and *Staphylococcus* compared to the metal/glass surfaces, which had a higher abundance of *Leuconostocaceae*, *Comamonadaceae*, *Enterobacteriaceae*, and *Novosphingobium* (Fig. 4B).

### Metagenomics-based taxonomic and functional analyses.

**FIG 5:**
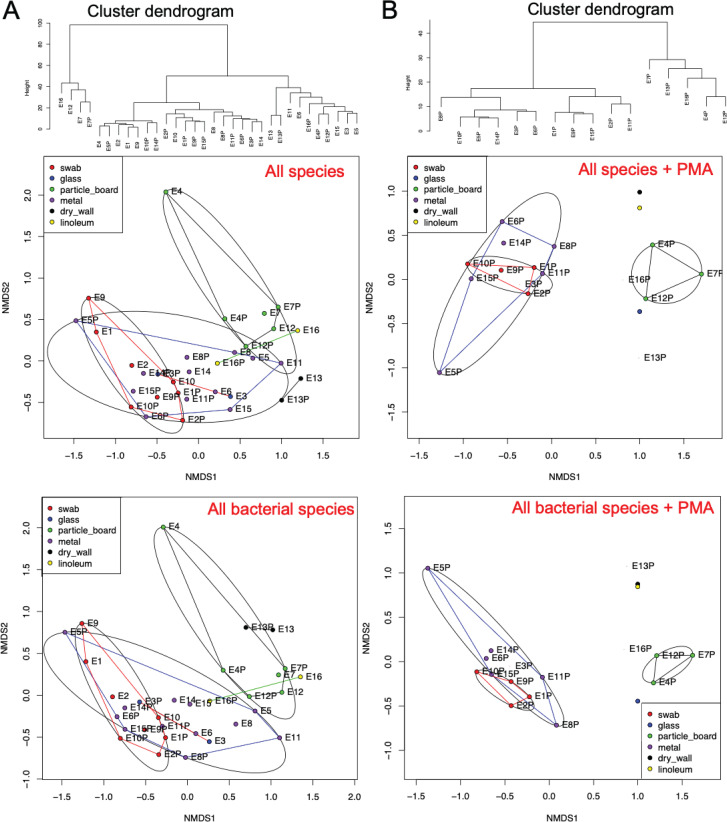
Metagenomic sequencing analysis of the Analog habitat. Cluster dendrogram of Euclidean distances was performed on non-PMA-treated (A, top) and PMA-treated samples (B, top). NMDS ordination showing the 95% confidence interval ellipse based on Unifrac distances in the matrix of all microbial species (bacteria and fungi) from both non-PMA-treated (middle left panel) and PMA-treated (middle right panel) samples. Similar treatment was performed for all bacterial species and NMDS ordination plots are depicted for non-PMA-treated (bottom left panel) and PMA-treated (bottom right panel) samples. The samples collected from various Analog habitat surfaces were indicated by various color circles.

Metagenomics-based taxonomic analysis was performed (in tandem with 16S rRNA amplicon sequence analysis) to characterize the microbial populations across the sample locations. Bacteria were the most dominant domain present in the surface samples from the Analog habitat, accounting for 94% of all characterized sequences. Eukaryotes made up 5.6% of the sequences, with most of these sequences belonging to the fungi and mammals. The remaining sequences were below 0.3% and are thus not discussed in detail. No archaeal signatures were observed in the metagenomic data set. There were no microbial community differences observed between the samples that were not treated with PMA (i.e., dead cells or naked DNA; Fig. 5). However, when samples were treated with PMA (live/intact cells), microbial community differences were apparent between locations, specifically between the LDP and metal/glass surface samples (Fig. 5). Additionally, the control group related closely with the metal/glass surface samples.

Through shotgun metagenomics, 136 genera were observed in non-PMA-treated samples, of which 5 and 75 genera were not identified in the PMA-treated samples of particle board and metal surfaces, respectively. Similar to the 16S rRNA amplicon sequencing, shotgun metagenomic sequencing identified comparable microbial populations at the genus level with the predominance of *Brevibacterium* (53.6%), *Brachybacterium* (7.8%), *Pseudonocardia* (9.9%), *Mycobacterium* (3.7%), and *Staphylococcus* (2.1%). Analogous to bacterial diversity, the fungal richness of non-PMA-treated (total) samples decrease compared to PMA-treated (viable) samples. Only two fungal phyla were detected that belong to *Ascomycota* and *Basidiomycota* (Fig. S3A). The frequencies of the fungal operational taxonomic units (OTUs) at the genus level for various surface samples are presented in Fig. S3B. The most dominant *Aspergillus* and *Penicillium* in the metagenome analyses were also found to be most frequently isolated during cultivation process (Fig. S2 and S3B).

10.1128/mSystems.00367-20.3 FIG S3 Relative abundances of microbial taxa (genera to higher taxonomy level [A] and fungal genera [B]) identified in PMA-treated and non-PMA-treated samples of the Analog habitat were visualize by bar plots. Download FIG S3, PDF file, 0.1 MB. Copyright © 2020 Malli Mohan et al. 2020 Malli Mohan et al. This content is distributed under the terms of the Creative Commons Attribution 4.0 International license.

### Functional pathway analysis.

**FIG 6:**
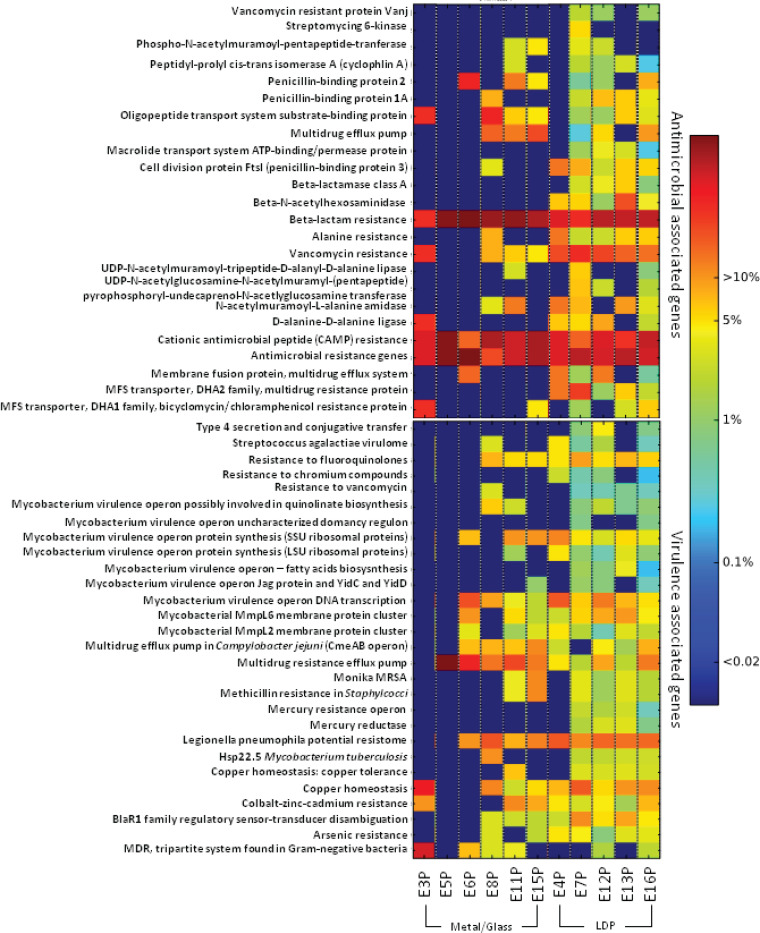
Metagenomic sequencing analysis of bacteria of the Analog habitat. Heat map showing the relative abundance of each antimicrobial-associated gene (top) and virulence-associated genes (bottom) detected in each sample collected.

Metagenomic sequencing gives the ability to inspect genomic and metabolic capability of the microbial community members. To examine the presence of a functional gene, sequence reads from all samples were mapped to individual microbial genes, which were then assigned to KEGG ortholog pathways. The microbial population within the LDP samples exhibited enrichment over other samples for pathways associated with general microbial proliferation, including nucleotide and amino acid metabolism, signal transduction, and cell motility and communication (Fig. S4). The gene families for carbohydrate utilization, amino acids and derivatives, and protein and RNA metabolism were found across all sampling locations. Additionally, presence and relative abundance of antimicrobial resistance genes (AMR) were annotated in the Comprehensive Antimicrobial Resistance Database (CARD). About 24 antimicrobial resistance gene families were identified throughout the Analog habitat, including resistance to aminoglycosides, beta-lactams, clindamycin, fluoroquinolones, lincosamide, streptomycin, and tetracycline (Fig. 6). In general, a larger proportion of AMR-associated sequences were observed in the LDP samples relative to the metal surfaces. A range of AMR categories were identified, including resistance to aminoglycosides, beta-lactams, clindamycin, fluoroquinolones, lincosamide, streptomycin, and tetracycline. Overall, beta-lactam resistance (21%) and cationic antimicrobial peptide (CAMP) resistance genes (8%) showed higher abundance across all locations than other AMR genes. In addition to AMR analysis, microbial genes were also screened for virulence factors using the Virulence Factors Database (VFDB; 2017). Sequence reads corresponding to virulence genes were binned into functional categories that combine genes contributing to similar mechanisms of virulence, including efflux proteins, transposases, methylases, and resistance to a range of antibiotics (Fig. 6). There is substantial overlap between genes annotated as conferring AMR and those implicated in virulence; thus, a proportion of the microorganisms present that contain virulence-associated genes are associated with resistance as well.

10.1128/mSystems.00367-20.4 FIG S4 Heat map showing the relative abundance of metabolism-associated genes detected in each sample collected. Download FIG S4, PDF file, 0.1 MB. Copyright © 2020 Malli Mohan et al. 2020 Malli Mohan et al. This content is distributed under the terms of the Creative Commons Attribution 4.0 International license.

## DISCUSSION

Extensive microbial diversity studies have been conducted in various Earth-based analog environments (e.g., Mars 500, and multiple samplings of ILMAH) as well as aboard the ISS, all of which relied on standard swabs and wipe kits (11, 13–15, 26). Even though NASA has used similar cotton, rayon, and polyester swab devices for sampling spacecraft surfaces and associated environments since the 1970s (16, 17), the use of these standard swabs and wipe kits may prove insufficient for valid sampling outside of the ISS and other future spacecraft to monitor and prevent forward contamination. Published reports comparing the collection of microbial samples from various surfaces using wipes, sponges, vacuum-based samplers, and a variety of swab head materials for culture-based target organisms has demonstrated large variability in microbial recovery outcomes (18, 19, 21, 22). Of the devices and materials tested, macrofoam was demonstrated to be the superior material with respect to both collection and recovery efficiency. Macrofoam swabs were demonstrated to collect ≥30% more spores compared to rayon or polyester swabs, even at low biomass testing conditions (21). In multiple assessments, including cultivation-based investigations and rRNA gene-based qPCR analysis, macrofoam was found to outperform polyester and cotton materials at collecting microbial cells and spores across numerous surface types (31). Along with having inferior collection rates compared to macrofoam, these other swabs are also more reactive to temperature and pressure changes that would occur while sampling surfaces outside the ISS, which could lead to sample loss (29). The inadequacy of the standard swabs is compounded by the difficulty of using swabs or wipes while astronauts are wearing large, bulky EVA gloves. To overcome these hinderances, a team of NASA engineers and scientists have developed a unique microbial EVA swab kit that combines the macrofoam material and a swab holder designed to function both with astronauts wearing EVA gloves and also interface with robotic manipulators (29). Thus, the use of the new EVA swab kits during sampling should facilitate a more accurate account of the microorganisms on the surfaces being tested while also allowing for more versatility in sampling locations.

Of the 10 cultivatable bacteria identified, species associated with pathogenicity, toxin production, biofouling, and biocorrosion were documented. The dominant cultivable bacterial isolates were spore-forming *Bacillus*, with the most common culturable bacterial isolate across the Analog habitat being Bacillus pumilus (∼48%). B. pumilus was isolated from all but the E3 sample site (center of the view port). Strains of *B. pumilus* have been shown to be capable of becoming opportunistic pathogens in immunocompromised patients (32), through the production of toxin (33), while other strains have been implicated in the biocorrosion of galvanized steel (34). The second most common bacterial isolate was Bacillus cereus, making up ∼15% of the bacterial population. Strains of this species have previously also been characterized as a pathogen (35), an opportunistic pathogen (35), and toxin producer (36). Also similar to *B. pumilus*, some B. cereus strains have been shown to cause corrosion (37) and water system fouling (37, 38). As members of the *Bacillus* genus are common inhabitants of soil and dust, they were likely to be cultured from the LDPs of the Analog habitat. Because *Bacillus* readily forms spores that are difficult to extract DNA from, they may have previously escaped detection in some earlier studies of surface samples (39–41). Likewise, in this study, sequencing methods again did not retrieve sequences of some spore formers that were cultured. However, spore formers, including members of the genera *Bacillus*, *Paenibacillus*, *Virgibacillus*, and *Oceanobacillus* were detected via cultivation. Also, both cultivation-dependent and sequencing methods confirmed the presence of *Staphylococcus* species in the Analog surface samples. *Staphylococcus* species are normally associated with human skin and may cause infections under certain situations in immunocompromised patients (42). Similar to the ISS, the closed nature of the Analog habitat makes it likely that the majority of these bacteria originate on the skin of crew members and fall off with the shedding of dead skin cells (13).

Of the nine cultivatable fungal lineages, we found a variety of species with related isolates that have previously been associated with pathogenicity, mycotoxin production, biofouling, and biocorrosion. Members of the *Aspergillus* genus were the dominant cultivable fungi, making up five of the nine species isolated, with the most common culturable fungal isolate across the Analog habitat being Aspergillus sydowii (36%). A. sydowii was found in all locations but E8 and E16 (entry table and trash can storage, respectively). A. sydowii was reported to be a marine pathogen of seafans (43), produce mycotoxin (44), and also associated with biocorrosion damage aboard the ISS (45). The second most common fungal isolate was Aspergillus tubingensis, making up ∼15% of the fungal population isolated during this study. Although a few strains of A. tubingensis have previously been identified as being rare opportunistic pathogens (46) through the production of mycotoxin (47), A. tubingensis is more well-known for its ability to degrade plastic (48). Multiple microorganisms and microbial processes have been implicated in the biodegradation and biofouling of human-made materials and structures (49). In this study alone, 7 of the 19 microbial isolates (fungal and bacterial) have been previously associated with biofouling or biocorrosion, while 13 of the 19 microbial isolates have been previously associated with pathogenicity (at minimum opportunistic) and or toxin/mycotoxin production; however, the previous association of these select isolates with disadvantageous traits does not indicate that these microbes are performing these processes in the Analog habitat. Future mitigation strategies should be adopted to prevent any buildup of excess moisture in the Analog habitat. There has been no evidence that any isolate cultured from the Analog habitat has led to harm of the crew or the habitat, and there has been no indication that any of the microbes isolated here pose a medical threat to the crew.

Several studies have been reported on the microbial composition of Analog habitat environments used as proxies for future human exploration using gene-targeted amplicon sequencing of microbial populations. One such study, the ILMAH, exhibited high abundance of *Staphylococcaceae*, *Corynebacteriaceae*, *Caulobacteraceae*, *Pleosporaceae*, and *Sporidiobolaceae* (11). A similar closed system, Mars 500, showed a high abundance of sequences of *Corynebacteriaceae*, *Burkholderiaceae*, and *Staphylococcaceae* (26). The submerged Analog habitat’s cultivable microbial composition was dominated by *Bacillus* (72%) and *Staphylococcus* (15%), indicating that this submerged analog environment differs from terrestrial analog environments (ILMAH and Mars 500).

In comparing the OTU assignments generated from 16S rRNA analysis (Fig. 3) and OTUs generated from metagenomic analysis (Fig. 2), it is clear that when nonviable cells are removed by treatment with PMA, the LDP surface biomes and the glass/metal biomes form separate groupings. This is in contrast to when there is no treatment and all cells (viable and nonviable) are compared, and a large poorly defined group is formed. This suggests that there is a shared background of nonviable cells across the habitat and that the different niches of LDP and the glass/metal are selecting for different live/intact microbial communities. The dendrogram in Fig. 5 closely corroborates this trend, with the exception of location 13 grouping with the LDP group. Additionally, the controls in Fig. 2 to 4 group closely with the glass/metal surface. It is unclear whether this grouping is an artifact due to the low cell numbers in both the control and metal/glass groups, and if so, what variables have led to the microbial population of the metal/glass to be so low. These could range from more stringent cleaning regimes, innate antimicrobial properties of the materials, or simply a lack of contact with microbe-containing objects and/or people.

Our metagenomic approach revealed relative abundance of metabolic pathways, virulence factors, and AMR genes (50, 51). The AMR gene categories specifically relating to the transformation proteins (penicillin binding protein [PBP]), an efflux pump (membrane fusion protein) similar to the ISS metagenomic AMR profile were abundant (Fig. 5). Additionally, *Mycobacterium* virulence operon, metal resistance mechanisms such as cobalt-zinc-cadmium resistance and copper homeostasis, were identified which are also similar to the ISS metagenomic profile (15).

### Conclusions.

The objective of this study was to determine the microbial ecology of a submerged spacecraft analog habitat using cultivation and molecular methods. The EVA swab kits employed were able to collect viable cells, cultivable microorganisms, and sufficient genetic material for 16S rRNA, ITS, and metagenomic shotgun sequencing methodologies. This study demonstrated collection of microorganisms from a variety of surfaces that ranged from smooth glass to rough and irregular materials. Based on the results, it is recommended that highly textured and absorbent particle board should not be used in closed human habitats or in current or future spacecraft, as these harbor more microorganisms. These findings are supported by previous studies that demonstrate that rough irregular and spongey surfaces can protect large microbial loads (52) and that it is much easier to remove cells from smoot homogenous surfaces (53). Finally, numerous microbes isolated from the Analog habitat have also been previously found aboard the ISS and/or MIR stations (45, 54, 55). This similarity, along with the habitat’s mix of isolates related to potentially opportunistic pathogens and biocorrosion-associated microbes, indicates that the closed Analog habitat may be the ideal location to test future microbial monitoring and microbial mitigation techniques as NASA begins to build and design new space architecture.

## MATERIALS AND METHODS

### EVA swab sample kit preparation and sample collection.

**FIG 7:**
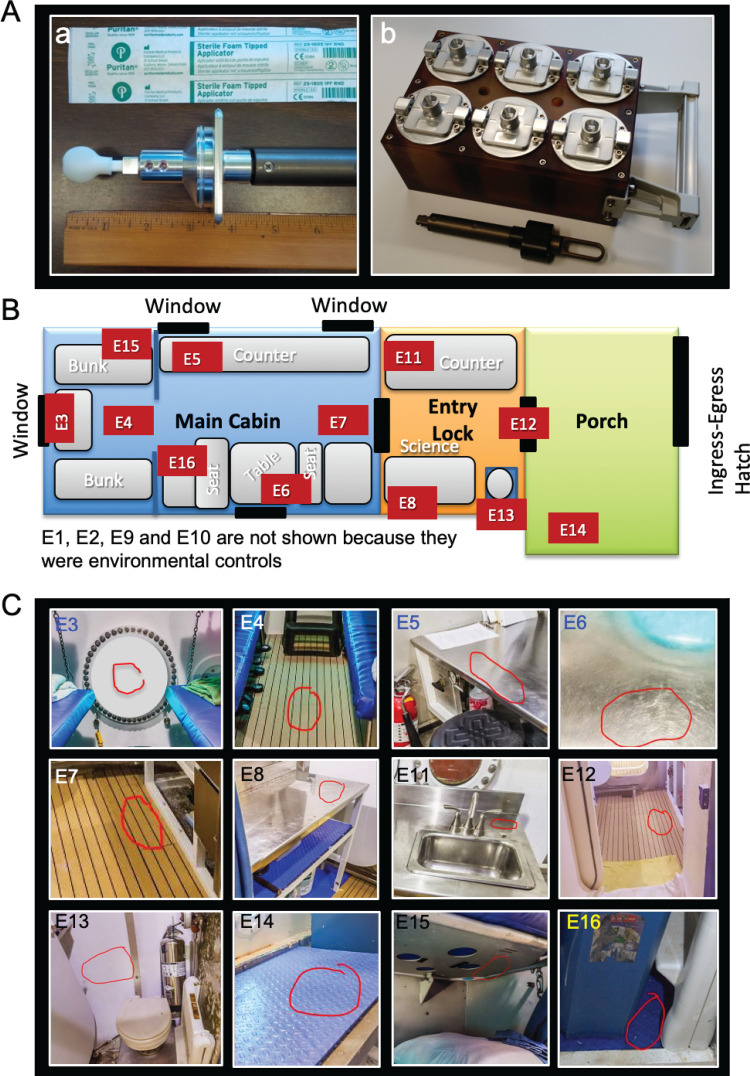
Locations and sampling tool kit feature for surface sampling. (A) Cantilever swab tool kit storage canister box (a) and swab head attached to the cantilever tool kit (b). (B) Two-dimensional (2D outline) of the Analog habitat and sampling locations (E1 to E16). (C) Photographs of Analog habitat sampling locations. The red circles indicated the areas where the samples were collected.

The EVA swab head is shown in Fig. 7Aa, and the six EVA swab kits holder (caddy) are depicted in Fig. 7Ab. Sample kit sterilization and assembly were performed at JSC. Each sample canister (assembled with filter and ball plungers) and swab end effector assembly was placed into separate autoclave bags. Bagged components were placed into a Steris LV 250 laboratory steam sterilizer and sterilized using a gravity cycle of 45 min at 121°C at 103.4 kPa (15 lb/in^2^). Note that neither the sample caddy itself nor the tool handle were autoclaved. Bagged components were allowed up to 1 h of cool-down time at approximately 22°C for safe handling. Following autoclaving, bagged components were transferred to a Labconco horizontal clean bench (model no, 36100000, ISO class 5). With the commercial swab inside its sterile packaging, the swab stem was cut to the optimal length (approximately 6.0 cm [2.4 in]) using sterilized scissors, ensuring that the swab head remained inside its packaging until the final assembly step. The cut end of the swab was then inserted into the end effector slot, and set screws were tightened to hold the swab in place. Sterile packaging was removed from the swab head immediately before inserting each swab assembly into its sterile container. Each container/swab assembly was then mounted into the tool caddy, which was placed into storage until sampling. During swab assembly, technicians wore sterile gloves, and both the gloves and assembly tools (Allen wrench, scissors, and forceps) were sprayed with ethanol surface disinfectant. All parts were handled either with sterile forceps or the autoclave bags, with no contact between the gloves and tool areas that must remain sterile. After assembly, the EVA sample kits were transported to the test site packed inside hard-sided storage cases. Once at the test site, the Analog crew were briefed on tool usage and given an opportunity to practice with a spare handle and sample container assembly.

Surface areas (25 cm^2^) were sampled with EVA kit swabs, which were premoistened with sterile phosphate-buffered saline (PBS) just prior to sampling. Surfaces were first sampled in a unidirectional horizontal manner while holding the swab at approximately a 30° angle to the surface. Swabs were rotated (ca. 120°) to present an area of the swab head that had not previously contacted the surface, and coupons were sampled in a unidirectional vertical manner. Finally, swabs were once again rotated (ca. 120°), and surfaces were sampled in a unidirectional diagonal manner. Surface samples were collected from 12 different locations across the Analog habitat using the EVA microbial swab kit. To serve as controls, two swabs were removed from kits and exposed to the Analog habitat atmosphere before being placed back into the container, and another two were left in the container. Both control swabs and surface swabs were processed in tandem. A schematic representation of the Analog habitat sampling locations and the corresponding images are shown in Fig. 7B and C. In this study, locations were categorized into three types, control (*n* = 4), linoleum, dry wall, and particle board (LDP; *n* = 5), and metal/glass (*n* = 7). Samples 1 through 8 were collected approximately 3 days of crew occupation into the analog mission; samples 9 through 16 were collected 5 days later. Both sets of samples were collected in the late afternoon/early evening. Control samples (E1 and E9) remained inside their sample canisters. An additional two control samples (E2 and E10) were removed from their canisters flagged for ∼60 s inside the habitat and then replaced without the swab head touching any surface. Immediately after sampling, the EVA microbial swabs were kept in a refrigerator and subsequently shipped at 4°C for overnight delivery to JPL, and samples were processed within a few hours of reaching JPL. The total time taken from sample collection to the start of sample processing, including transportation, was less than 36 h. The Analog habitat sampling locations are illustrated in Fig. 7B and C, and their associated metadata are summarized in Table 1, describing each sample location and other characteristics of the sampled surfaces.

### Test facility and control.

NASA’s Analog mission involved a crew of six astronaut candidates and trainees residing in the habitat for 16 days, with periodic scuba excursions to the surrounding environment to perform simulated EVA spacewalks. The size of the Analog habitat is 45 feet long, with a 13-foot diameter. The submerged Analog mission habitat was comprised of three sections (Fig. 7B). A 40-m^3^ main cabin area which contained the crew living quarters for up to six. The 14-m^3^ entry lock that included science and hygiene areas, and the 20-m^3^ porch, which allowed crew to ingress/egress between the habitat and external environment (Fig. 7B). The main cabin was controlled to a standard atmosphere (21% oxygen) at 101.3 kPa (14.7 lb/in^2^), provided by an air compressor. Relative humidity ranged from 70% to 100%, and temperatures ranged from 24°C to 28°C; crews typically preferred to set the thermostat on the warmer end of the temperature range. Carbon dioxide was chemically scrubbed from the atmosphere inside the cabin.

### Sample processing.

Sample canisters (with swab assemblies still in place) were removed from the sample caddies and placed into a hard-sided shipping container, with the sample canisters secured in foam packing. Samples were then shipped via air to the Jet Propulsion Laboratory, and sample processing took place in a clean room. Each EVA swab was aseptically removed from the lock and transferred to a 50-ml sterile falcon tube containing 15 ml of sterile PBS (pH 7.4). The falcon tube with the EVA swab was shaken for 2 min, followed by the concentration with a concentrating pipette (Innova Prep, Drexel, MO) using 0.45-μm hollow fiber polysulfone tips (catalog no. CC08022) and PBS elution fluid. Each sample was concentrated to 1 ml and made up to 5 ml using sterile PBS. A 200-μl aliquot was combined with 1.8 ml of sterile PBS (up to 10^−1^) to estimate cultivable population as well as ATP content (Kikkoman Corp., Noda, Japan) as described previously (56). Three milliliters of sample was split into two 1.5-ml aliquots. One aliquot was treated with PMA to assess viability (57), while the second aliquot was handled similarly but without the addition of PMA. The 18.25 μl of 25 μM PMA was added to the PMA treatment samples and then incubated for 5 min at room temperature in the dark, followed by 15-min exposure to the activation system (PMA LED device; Biotium, Hayward, CA). The samples were then split in half again (0.75 ml per tube) and transferred to bead beating tubes containing Lysing Matrix E (MP Biomedicals, Santa Ana, CA). One half of PMA-treated and non-PMA-treated samples were individually subjected to bead beating for 60 s using a vortex sample holder (MO Bio, Carlsbad, CA). The bead-beaten portion and the unprocessed aliquot were combined, followed by DNA extraction with the Maxwell 16 automated system (Promega, Madison, WI), in accordance with the manufacturer’s instructions. Maxwell Control (MC) was an additional cartridge run concurrently with each sample set to account for any contamination during the DNA extraction (58, 59). The extracted DNA was eluted in 50 μl of water and stored at −20°C until further analysis.

### Estimation and identification of cultivable microbial population.

For the cultivation experiments, 100 μl of each dilution was plated on Reasoner’s 2A agar (R2A), potato dextrose agar (PDA) with chloramphenicol (100 μg/ml), and blood agar (BA) (Hardy Diagnostics, Santa Maria, CA). The R2A and PDA plates were incubated at 25°C for 7 days, and the BA plates were incubated at 35°C for 2 days, at which time CFU were calculated. Whenever possible, several colonies depicting distinct morphologies were picked from each plate from each sampling location. The isolates were then archived in the semisolid R2A or PDA slants (agar medium diluted 1:10) and stored at room temperature. Once a culture was confirmed to be pure, DNA was amplified during a colony PCR, or it was extracted with either the UltraClean DNA kit (MO Bio, Carlsbad, CA) or the Maxwell Automated System (Promega, Madison, WI). To identify bacterial isolates, we PCR amplified with the 16S rRNA primer pair 27F (5′-AGA GTT TGA TCC TGG CTC AG-3′) and 1492R (5′-GGT TAC CTT GTT ACG ACT T-3′) (60, 61). The PCR cycle conditions were as follows: (i) denaturation at 95°C for 5 min; (ii) 35 cycles, with 1 cycle consisting of denaturation at 95°C for 50 s, annealing at 55°C for 50 s, and extension at 72°C for 1 min 30 s; (iii) a final extension at 72°C for 10 min. To identify fungal isolates, we amplified the fungal variable sized partial internal transcribed spacer (ITS) region with the primer pair ITS1F (5′-TTG GTC ATT TAG AGG AAG TAA-3′) (62) and Tw13 (5′-GGT CCG TGT TTC AAG ACG-3′) (63). The PCR conditions were as follows: (i) initial denaturation at 95˚C for 3 min; (ii) 25 cycles, with 1 cycle consisting of 95°C for 50 s, annealing at 58°C for 30 s, and extension at 72°C for 2 min; (iii) a final extension at 72°C for 10 min. The amplification products were inspected by gel electrophoresis in 1% agarose gel and 1.5-kb molecular weight size marker. The amplicon sequencing was performed by Macrogen (Rockville, MD, USA) using 27F and 1492R universal primers for bacteria, and ITS1F and Tw13 universal primers for fungi. The sequences were assembled using SeqMan Pro from DNAStar Lasergene Package (DNASTAR Inc., Madison, WI). The bacterial sequences were searched against EzTaxon-e database (64), and the fungal sequences were searched against the UNITE database (65). The identification was based on the closest percentage similarity (>97%) to previously identified microbial type strains.

### ATP assay.

A bioluminescence assay was performed to determine the total ATP and intracellular ATP from all samples using the CheckLite HS kit (Kikkoman, Japan), as described previously (56). Briefly, to determine total ATP (dead and viable microbes and other organics), sample aliquots were combined with an equal volume of a cell lysing detergent (benzalkonium chloride) and incubated at room temperature for 1 min prior to the addition of a luciferin-luciferase reagent. The sample was mixed, and the resulting bioluminescence was measured with a luminometer (Kikkoman). For intracellular ATP measures of intact microbes, a 1/10 volume of an ATP-eliminating reagent (apyrase, adenosine deaminase) was added to the sample and allowed to incubate for 30 min to remove any extracellular ATP. After extracellular ATP removal, the assay for ATP was performed (as described above) while running sterile PBS in tandem as a negative control. One relative luminescence unit (RLU) (the unit of ATP measurement) equates to approximately 1 CFU (56).

### qPCR assay.

Following DNA extraction with the Maxwell Automated system, quantitative PCR (qPCR) targeting both the bacterial 16S rRNA gene and the fungal internal transcribed spacer region was performed with SmartCycler (Cepheid, CA) to quantify the microbial burden. Primers targeting the 16S rRNA gene were 1369F (5′-CGG TGA ATA CGT TCY CGG-3′) and modified 1492R (5′-GGW TAC CTT GTT ACG ACT T-3′) (66). Primers targeting the ITS region were NS91 (5′-GTC CCT GCC CTT TGT ACA CAC-3′) and ITS51 (5′-ACC TTG TTA CGA CTT TTA CTT CCT C-3′) (67). Each 25-μl reaction mixture consisted of 12.5 μl of 2× iQ SYBR green Supermix (Bio-Rad, Hercules, CA), 1 μl each of forward and reverse oligonucleotide primers (10 μM each), and 1 μl of template DNA (templates included PMA-treated and non-PMA-treated samples). Each sample was run in triplicate, and the average and standard deviation were calculated based on these results. The reaction conditions were as follows: a 3-min denaturation at 95°C, followed by 40 cycles of denaturation at 95°C for 15 s, and a combined annealing and extension at 55°C for 35 s. The numbers of gene copies in the samples were determined by the threshold cycle (*C_T_*) values of the samples and the *C_T_* values of the standard curve, which is generated automatically by the instrument. The standard curve was generated using serial dilutions (10^8^ to 10^2^) of synthetic Bacillus pumilus SAFR-032 16S rRNA gene. The qPCR efficiency as generated automatically by the instrument was ∼ 98% for each run. DNase/RNase-free molecular-grade distilled water (Promega, Madison, WI) was used as the negative control in each run. The negative controls had *C_T_* values that were greater than 37.

### 16S rRNA gene amplicon sequencing.

DNA from these samples was amplified using ∼100 pg of genomic DNA (gDNA) in triplicate within 25-μl volume reactions using Platinum Hot Start PCR master mix (catalog no. 13000012; Thermo Fisher ) and Earth Microbiome Project standard Golay-barcoded primers of the 16S V4 region, 515fB (5′-GTG YCA GCM GCC GCG GTA A-3′) and 806rB (5′-GGA CTA CNV GGG TWT CTA AT-3′) (with expected amplicon size ∼291 bp) as described in the earthmicrobiomeproject.org at 94°C for 3 min, 35 cycles with 1 cycle consisting of denaturion at 94°C for 45 s, annealing at 50°C for 60 s, and extension at 72°C for 90 s, followed by a final extension step of 72°C for 10 min (68–71). Triplicate reactions were then pooled into a single tube, and quality was assessed. The amplicons were run on a 2% agarose gel and quantified using PicoGreen to assess quality and relative quantity. All samples were pooled in equal volumes into a single tube and then processed through the MoBio PCR cleanup kit to remove excess primers. The final cleaned pooled DNA was then then sequenced on a HiSeq 2500 2 × 150 bp Rapid Run.

### 16S rRNA gene amplicon sequence processing.

Sequencing reads were demultiplexed with Illumina CASSAVA analysis software. Adapters were clipped, and reads with <20 bp were removed. Corresponding forward and reverse reads were stitched into longer fragments using FLASH (overlap, 10 bp; maximum mismatch, 0.25). Amplicons of samples and controls were further sorted by removing reads without barcodes, single reads (only one barcode) and barcode chimeras (different barcodes on 5′ and 3′ sites). The resulting reads were quality filtered for deep diversity analysis with QIIME at a phred score of q30, oriented 5′ to 3′, labeled, and additional quality filtered using default settings in QIIME (72). OTUs were checked for chimeric sequences via ChimeraSlayer and clustered at 97% similarity level, taxonomy was assigned with SILVA, and the determined phylogenetic tree was calculated (73, 74). The resulting rarefied OTU table served as a basis for alpha and beta diversity analyses. The barplots, pie charts, and MDS plots were all created in R (v. 3.3.1) using the Hmisc and vegan packages.

### Shotgun metagenome sequencing.

DNA libraries from the Analog habitat’s surface DNA samples were prepared for shotgun metagenome sequencing using the Nextera DNA Library Preparation kit from Illumina. The quality and fragment size of each library were assessed on the Bioanalyzer 2100 (Agilent). Separate adapters were added to the DNA from each library, normalized to 2 nM, pooled, denatured, and diluted to 1.8 pM according to the standard recommendations by Illumina. The HiSeq 2500 platform (Illumina) was used for sequencing, resulting in 100-bp paired-end reads.

### Metagenome sequence data processing.

Paired-end 100-bp reads were processed with Trimmomatic (75) to trim adapter sequences and low-quality ends, with a minimum Phred score of 20 across the entire length of the read used as a quality cutoff. Reads shorter than 80 bp after trimming were discarded. All reads were normalized across samples as recommended by Nayfach and Pollard (76). All 16 sampling locations and two treatments (PMA and non-PMA) were studied with a total of 32 metagenomic samples. High-quality filtered reads were clustered to respective taxonomic levels (domains through species) using the lowest common ancestor (LCA) algorithm provided by MEGAN6 (77) and normalized to perform a semiquantitative comparative analysis. Microbial diversity analyses were conducted on normalized reads (∼ 3.1 × 10^8^), and analyses were set to keep at least one unique read to minimize the loss of diversity in low-depth samples or for unique reads. BLAST hits of ≥20 amino acids and ≥90% similarity were collected and used for taxonomic and functional assignment.

### Taxonomic and functional assignment of shotgun metagenome sequences.

For lower downstream processing and visualization, the MEGAN6 metagenomics toolkit was used (78). The NCBI taxonomy database (79, 80), containing more than 6.6 × 10^5^ reference sequences, and the NCBI-NR protein sequence database, consisting of entries from GenPept, Swiss-Prot, PIR, PDB, and RefSeq, were used to assign taxonomic features to reads by using DIAMOND (81) and the weighted LCA algorithm of MEGAN6 (77). The identification of the reads to a taxon is not based on the genes only, but it is based on the comparison of the reads with the reference sequences deduced from the genomes of the curated NCBI taxonomy database (80). Briefly, taxonomic and functional binning of the metagenomic reads is conducted using MEGAN (82), with the following settings: minScore = 50, maxExpected = 0.01, topPercent = 10, and minSupportPercent = 0.01. The resulting taxon assignments are presented in this article. Functional analysis was conducted by mapping filtered DNA sequences against a reference database of all proteins within eggNOG (83), SEED (84), and KEGG databases (85). The search for translated DNA sequences was executed using DIAMOND, and hits that spanned ≥20 amino acids with ≥90% similarity were retained. In cases where one read matched these criteria against multiple proteins, only the protein or proteins (in the event of a tie) with the maximum bit score were considered. Pathways were analyzed by summing counts of KEGG orthologies for each pathway. Using different databases allowed a detailed view of reads defined by gene function consisting of a collection of biologically defined (i) subsystems, (ii) clusters of orthologous groups, and (iii) collection of metabolic pathways.

### Assignment of virulence and antimicrobial resistance.

Detected genes were screened for antimicrobial resistance and virulence factors using the Comprehensive Antimicrobial Resistance Database (CARD; 2.0.3) and the Virulence Factors Database (VFDB; 2017) (86, 87).

### Statistical analysis.

Hierarchical clustering using the ward2 algorithm, and heatmap2 were conducted in the R programming environment in conjunction with the vegan and compositions package, as was analysis of variance (ANOVA) for univariate analysis of data (88). Box graphs of CFU and qPCR data were plotted using Prism (version 5.0a). Significance (*P* < 0.05) between groups was tested by a one-way ANOVA using Prism. Statistical analyses of CFU, ATP, and qPCR assays were performed with Student’s *t* test in Prism (version 8). Barplots were created in R (Version 3.6.1) using the Hmisc package. NMDS plots, permutational multivariate analysis of variance (PERMANOVA) analyses, and pie charts were created in R using the vegan package.

### Ethics approval and consent to participate.

Because the purpose of this test was to characterize microorganisms on the Analog habitat, rather than humans residing in the closed system, the JSC Institutional Review Board ruled this study as “Exempt Certified.” No identifying information about the crew members of the Analog habitat will be published.

### Data availability.

The Illumina 16S amplicon sequencing data have been deposited in the European Nucleotide Archive (ENA) under accession number PRJEB39173. The metagenomics data have been deposited into the NCBI SRA under BioProject number PRJNA559397 and accession numbers SRX7029161 to SRX7029192. GeneLab data are courtesy of the NASA GeneLab Data Repository (GLDS#292). The 16S sequences of cultivable bacteria (MN581166 to MN581196) and ITS sequences of cultivable fungi (MT560281 to MT560319) have been deposited in NCBI GenBank.

10.1128/mSystems.00367-20.6 DATA SET S2 Table of reads of shotgun metagenome sequences summarized to the family level. Download Data Set S2, XLSX file, 0.1 MB. Copyright © 2020 Malli Mohan et al. 2020 Malli Mohan et al. This content is distributed under the terms of the Creative Commons Attribution 4.0 International license.
