# Supplementary material for: Microbiome and Metagenome Analyses of a Closed Habitat during Human Occupation
Source: mSystems. 2020 Jul 28;5(4):e00367-20. doi: 10.1128/mSystems.00367-20 (PMC7394354; doi:10.1128/mSystems.00367-20)

Supplemental Figure S1

Bacterial Culture Classification	Analog habitat sampling locations								Abundance
	E3	E4	E5	E6	E7	E12	E13	E16	
Bacillus pumilus		1	4	2	3	4	1	1	16
Bacillus velezensis							1		1
Bacillus subtilis						2			2
Bacillus cereus		1			1			3	5
Virgibacillus chiguensis								1	1
Brevibacterium caesai							1		1
Paenibacillus lemnae						1			1
Staphylococcus haemolyticus	3								3
Staphylococcus cohnii			1						1
Staphylococcus arlettae					2				2
Total No. Isolates/Location	3	2	5	2	6	7	3	5	33

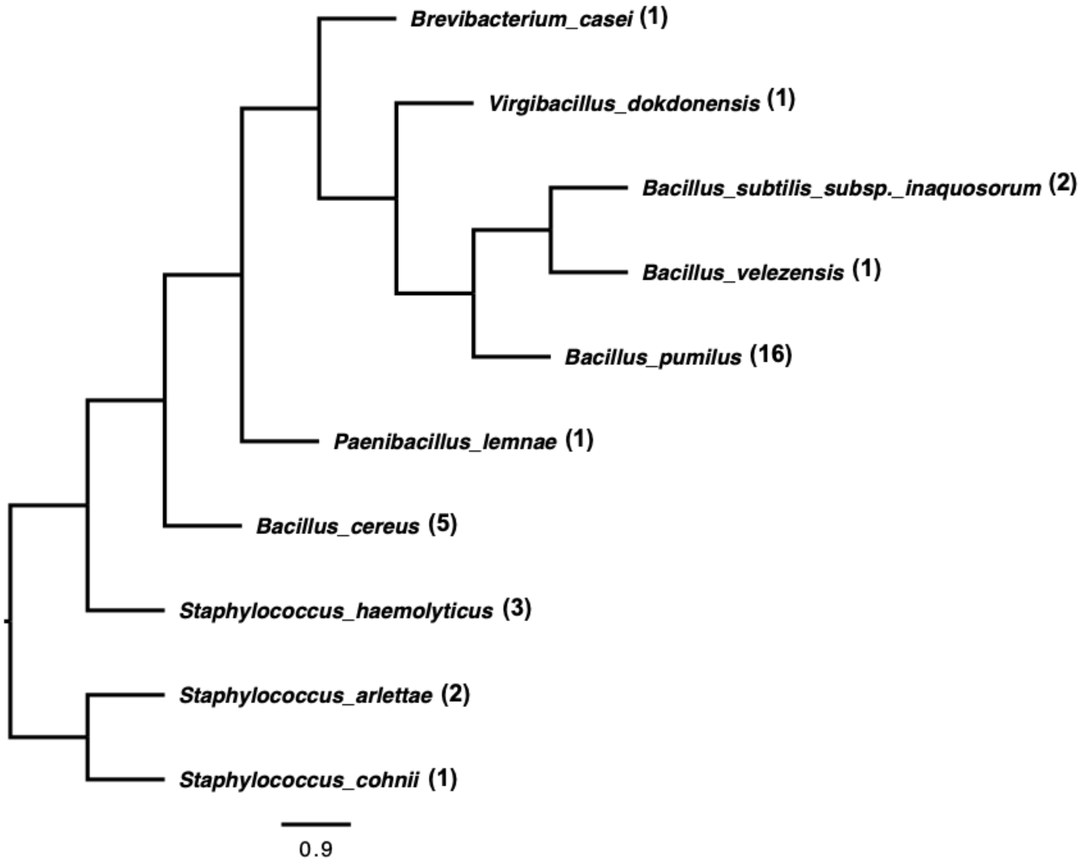

Supplement: FIG S1 [file mSystems.00367-20-sf001.pdf]
