# Supplementary material for: Microbiome and Metagenome Analyses of a Closed Habitat during Human Occupation
Source: mSystems. 2020 Jul 28;5(4):e00367-20. doi: 10.1128/mSystems.00367-20 (PMC7394354; doi:10.1128/mSystems.00367-20)

# Supplemental Figure S2

| Fungal Culture Classification | Analog habitat sampling locations | | | | | | | | | | | Abundance |
|---|---|---|---|---|---|---|---|---|---|---|---|---|
| | E3 | E4 | E5 | E6 | E7 | E8 | E11 | E12 | E13 | E15 | E16 | |
| *Aspergillus sydowii* | 3 | 2 | 1 | 2 | 4 | | 1 | 2 | 1 | 1 | | 17 |
| *Aspergillus silvaticus* | | | | | 1 | | | 2 | | | 1 | 4 |
| *Penicillium citrinum* | | 2 | | | | | | | | | | 2 |
| *Aspergillus tubingensis* | | | 3 | | | | | | 3 | | 1 | 7 |
| *Ascomycota sp.* | | 1 | 3 | | | 1 | 1 | | 2 | | | 8 |
| *Aspergillus flocculosus* | | | | | 2 | | | | | | 4 | 6 |
| *Penicillium meleagrinum* | | | | | | | | 1 | | | | 1 |
| *Aspergillus ivoriensis* | | | | | | 1 | | | | | | 1 |
| *Talaromyces radicus* | | | | | | 1 | | | | | | 1 |
| Total No. Isolates/Location | 3 | 5 | 6 | 2 | 7 | 3 | 2 | 5 | 6 | 1 | 6 | **47** |

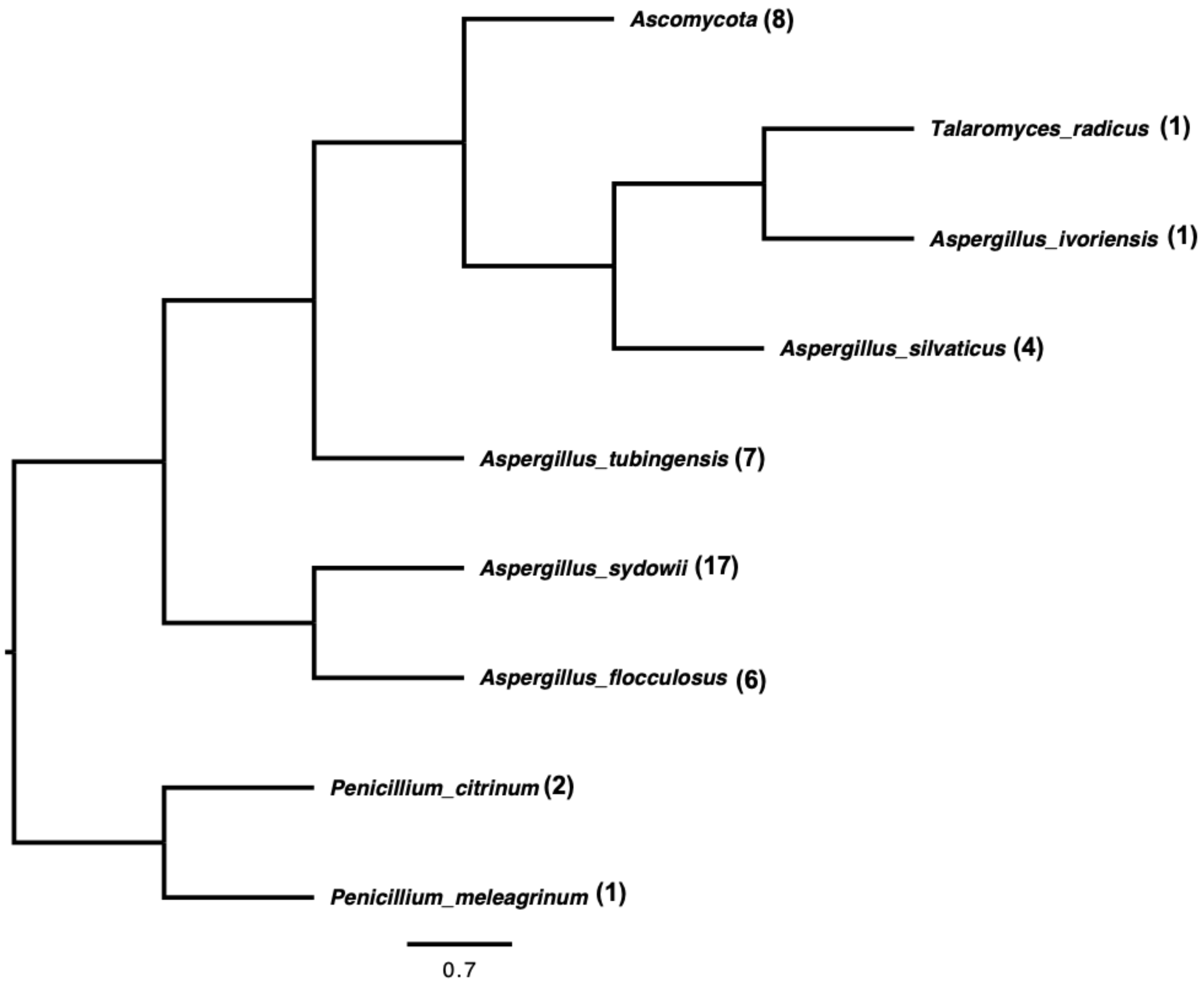

Supplement: FIG S2 [file mSystems.00367-20-sf002.pdf]
