# Supplementary figures and images for: Microbiome and Metagenome Analyses of a Closed Habitat during Human Occupation
Source: mSystems. 2020 Jul 28;5(4):e00367-20. doi: 10.1128/mSystems.00367-20 (PMC7394354; doi:10.1128/mSystems.00367-20)

# Supplemental Figure S3

A

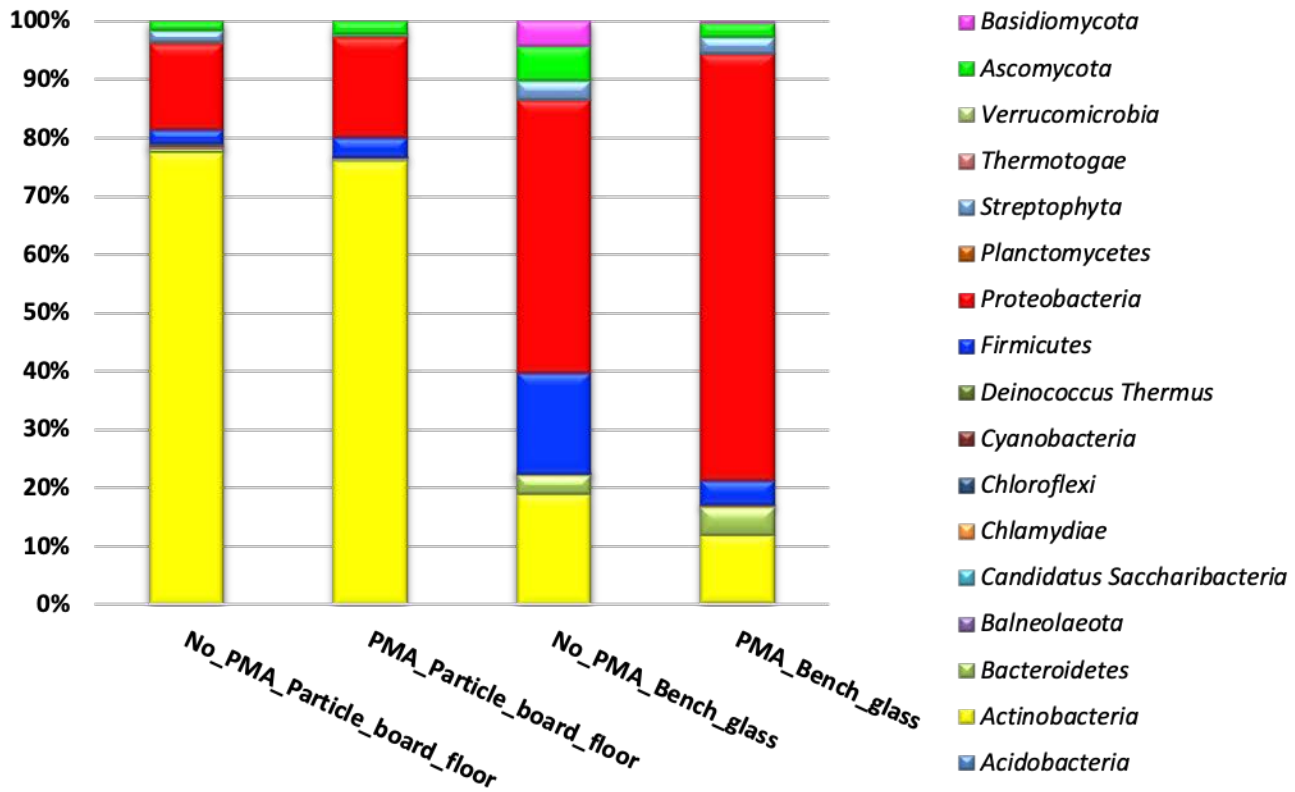

B

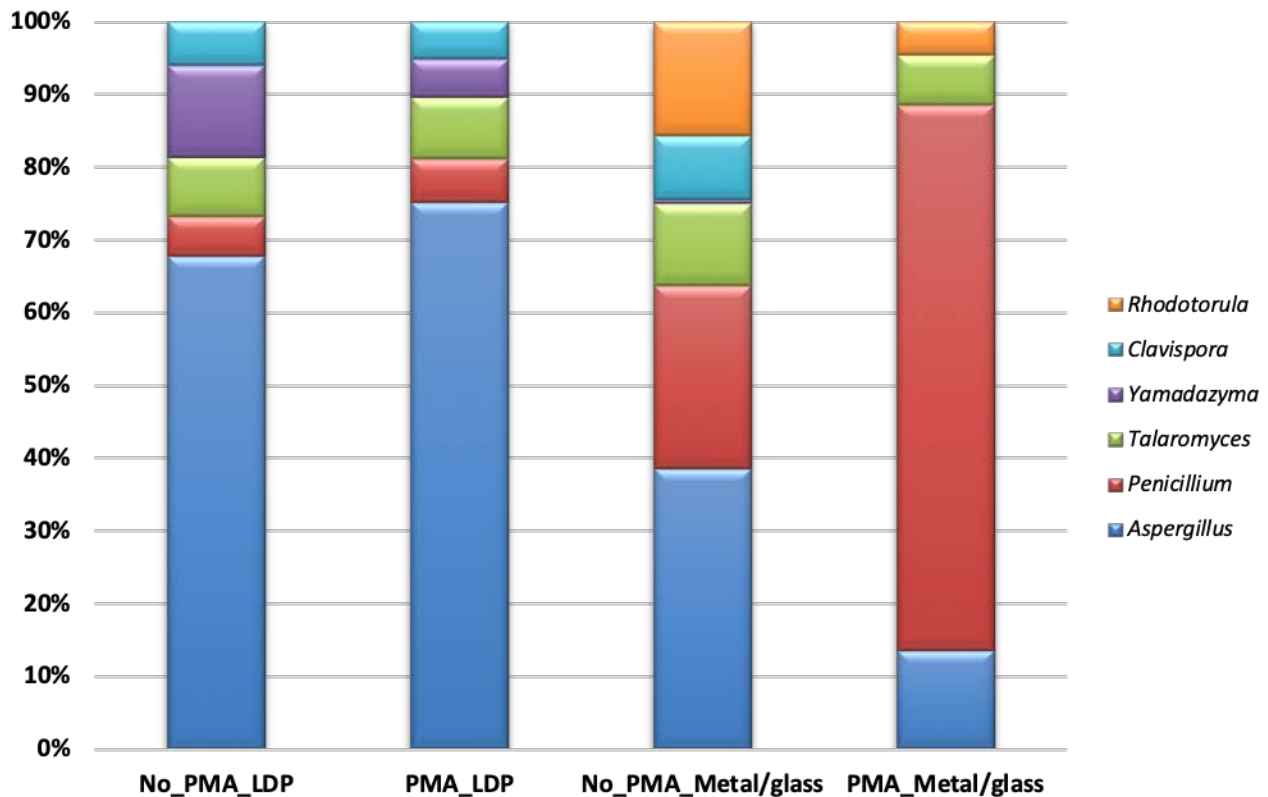

Supplement: FIG S3 [file mSystems.00367-20-sf003.pdf]

# Supplemental Figure S4

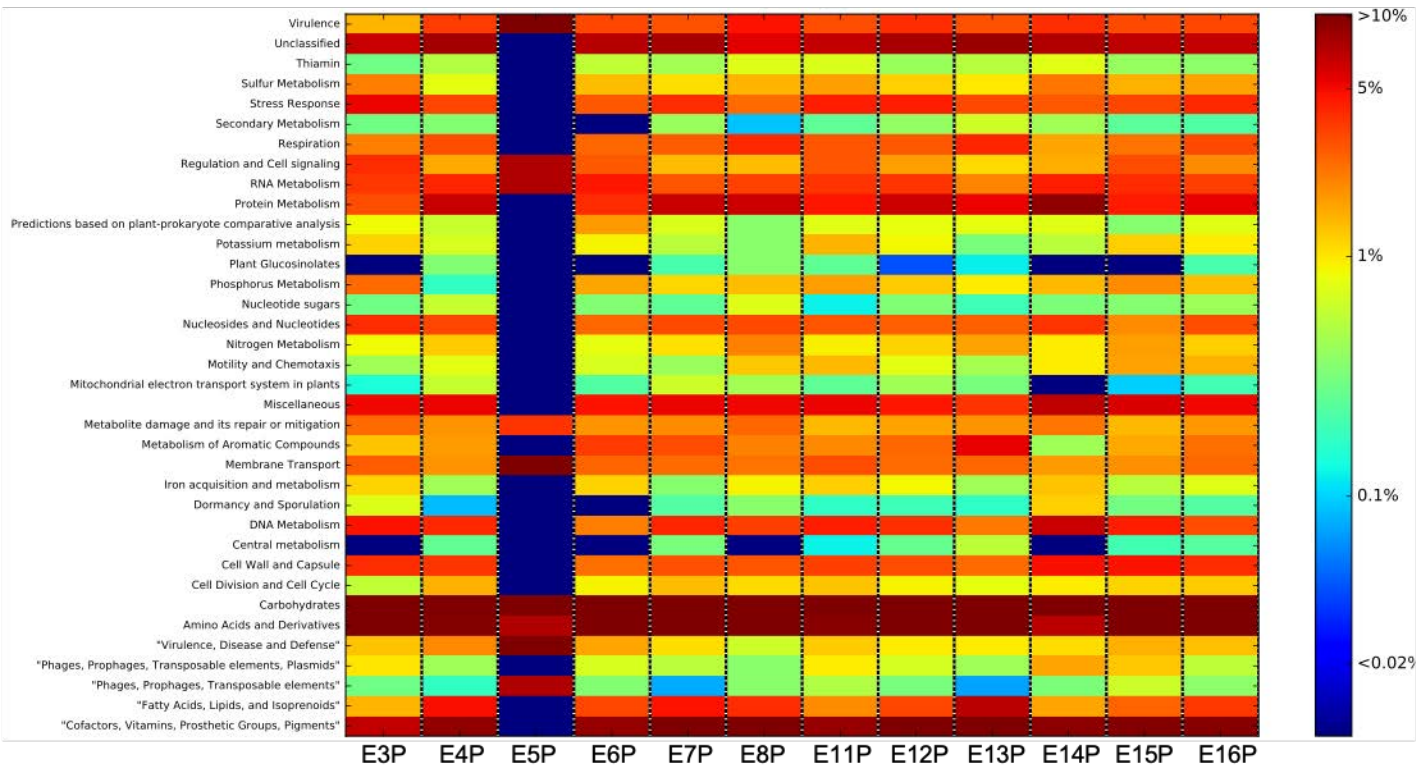

Supplement: FIG S4 [file mSystems.00367-20-sf004.pdf]
